# An Iterative Method for Problems with Multiscale Conductivity

**DOI:** 10.1155/2012/893040

**Published:** 2012-12-05

**Authors:** Hyea Hyun Kim, Atul S. Minhas, Eung Je Woo

**Affiliations:** ^1^Department of Applied Mathematics, Kyung Hee University, P.O. Box 446-701, Yongin, Republic of Korea; ^2^Samsung Electronics Co. Ltd., Suwon, Republic of Korea; ^3^Department of Biomedical Engineering, Kyung Hee University, P.O. Box 446-701, Yongin, Republic of Korea

## Abstract

A model with its conductivity varying highly across a very thin layer will be considered. It is related to a stable phantom model, which is invented to generate a certain apparent conductivity inside a region surrounded by a thin cylinder with holes. The thin cylinder is an insulator and both inside and outside the thin cylinderare filled with the same saline. The injected current can enter only through the holes adopted to the thin cylinder. The model has a high contrast of conductivity discontinuity across the thin cylinder and the thickness of the layer and the size of holes are very small compared to the domain of the model problem. Numerical methods for such a model require a very fine mesh near the thin layer to resolve the conductivity discontinuity. In this work, an efficient numerical method for such a model problem is proposed by employing a uniform mesh, which need not resolve the conductivity discontinuity. The discrete problem is then solved by an iterative method, where the solution is improved by solving a simple discrete problem with a uniform conductivity. At each iteration, the right-hand side is updated by integrating the previous iterate over the thin cylinder. This process results in a certain smoothing effect on microscopic structures and our discrete model can provide a more practical tool for simulating the apparent conductivity. The convergence of the iterative method is analyzed regarding the contrast in the conductivity and the relative thickness of the layer. In numerical experiments, solutions of our method are compared to reference solutions obtained from COMSOL, where very fine meshes are used to resolve the conductivity discontinuity in the model. Errors of the voltage in *L^2^* norm follow *O(h)* asymptotically and the current density matches quitewell those from the reference solution for a sufficiently small mesh size *h*. The experimental results present a promising feature of our approach for simulating the apparent conductivity related to changes in microscopic cellular structures.

## 1. Introduction

Electrical conductivity of a material is a measure of its ability to allow the movement of electric charge. Based on the material's atomic or molecular composition, electric charge may be either in the form of free electrons or ions. In a homogeneous saline solution, for example, there is ionic electric charge and its conductivity is determined by the total sum of the multiplication of concentration and the mobility of various ions presents in the solution [1]. The mobility of ions depends on the structural composition of the environment in which they are moving. A typical example is the movement of ions across the membrane of a cell in biological materials. The membrane itself is an insulator but it has pores which allow the flow of ions [2]. To better understand this bioelectric phenomenon a simple yet robust modeling method would be highly beneficial.

When a material is homogeneous, a simple way to measure its conductivity is to take the ratio of absolute value of the current density and electric field inside the material [1]. This is not so simple in biological tissues which are heterogeneous, consisting of cells, extracellular structures and fluids. To measure conductivity in a controlled way, we have to probe the material by injecting current and measure induced voltage or vice versa [1, 3, 4]. Other measurement methods include injecting the current or RF/acoustic magnetic field and measuring the induced magnetic field/flux density [5–10]. In this work, we consider the conductivity measurements using a technique called magnetic resonance electrical impedance tomography (MREIT). In MREIT, tissues are probed by externally injected currents and induced magnetic flux densities are measured using MRI scanner. An image reconstruction algorithm uses these measurements to reconstruct conductivity image of tissues [3].

The microscopic cellular structure affects the current flow pattern in biological tissues. The macroscopic conductivity measured by a current injection based probing method can be, therefore, understood as the apparent conductivity [11]. The apparent conductivity at a fixed macroscopic scale is determined as a congregation of microscopic effects within the tissue. Especially at low frequency, the membrane greatly affects a measured apparent conductivity value of the tissue at a macroscopic scale since the cellular membrane can be modeled as an insulating sphere with holes where ions can migrate [11]. An ability to model the microscopic effects within the tissue would be highly beneficial to better understand the nature of conductivity of biological materials.

Since we are interested in a macroscopic conductivity value, we need to understand how the microscopic structure influences the macroscopic conductivity measurement. To understand the meaning of a measured conductivity in relation with microscopic changes at the cellular membranes, we need quantitatively relate the apparent conductivity at the macroscopic scale with microscopic structural changes. Due to the scale difference, a numerical study may require a highly nonuniform mesh with a tremendously large number of elements resulting in a huge amount of computations and memory requirement, which may not be practically feasible.

There have been previous studies to address practical numerical methods for treating such a model heterogeneity [12–14]. In [13], finite difference methods were developed by correcting the finite difference stencils according to the conductivity discontinuity. Calculation of the correction term becomes quite complicated when a model with microscopic structure is considered. In [12], coarse finite element basis is built by solving the model problem in each coarse mesh with an appropriate boundary condition regarding the conductivity discontinuity. A discrete model is then built by using the coarse basis functions. This process can be understood as smoothing on the conductivity discontinuity. This approach still gives less accurate approximations than the work [13] and results in a ill-conditioned linear system depending on the heterogeneity of the conductivity. In [14], a practical discrete method was developed for simulating fluid-structure interaction by using two independent variables: Eulerian variable for the fluid on the background and Lagrangian variable for the immersed moving elastic body. After the separation, the interaction between the fluid and the elastic body is calculated by using a smoothed approximation to the Dirac delta function, where the Dirac delta function is used to model the location of the moving elastic body. The smoothed approximation is the force imposed by the elastic body on the fluid. After solving fluid equations with the exerting force, the location of the elastic body is updated by the fluid velocity. We refer the references therein for many successful applications of the immersed boundary methods; it is known to be the most practical discrete model for simulating a very thin elastic body.

A discrete model with certain smoothing on the microscopic structures will be more appropriate for our purpose than a very accurate discrete model. In this work, for a more practical method we propose an iterative method which employs a uniform mesh rather than a highly nonuniform mesh. We consider a model with a single cell and propose a new numerical method based on uniform meshes without much concern on the microscopic structures in the single cell. We first solve a simple model with a uniform conductivity and we then iteratively improve the numerical accuracy by updating the right-hand side of the simple model. The right-hand side is calculated by integrating the current solution over the single cell membrane, which could provide a certain smoothing effect similarly to that in [14] when a model with many of them is considered to study macroscopic properties related to changes in microscopic cellular structures. We note that at each iteration a simple model with the uniform conductivity is solved, thus any available fast solvers, that is, FFT (Fast Fourier Transform) [15] or multigrid methods [16], can be utilized to speed up the computing time. We analyze the convergence of the iterative method with respect to the contrast of the conductivity difference and the relative thickness of the cell membrane.

Our method is tested for a simple model with a single cell and then more complex models with many of small cells. These results are compared to reference solutions from COMSOL (COMSOL Inc., USA), where very refined nonuniform meshes are used to address high contrast of conductivity jump across the thin cellular membrane. The errors of voltage in *L* ^2^ norm asymptotically follow the first-order *O*(*h*) accuracy for the given mesh size *h* and the current density agrees quite well to that of reference solutions. To address capability of our method for capturing anisotropic cellular structures, we test a model with many cells, where holes are adopted to each cell membrane at various locations. Those results present a promising feature of our method for approximating macroscopic properties related to microscopic structural changes.

This paper is organized as follows. In Section 2, a model problem with multiscale structures, FE (finite element) discretization of the model problem, and an iterative method for solutions of the discrete model are described. In Section 2.5, convergence of the iterative method is analyzed related to the thickness of the cell membrane and the contrast of conductivity jump across the cell membrane. Numerical results are presented in Section 3. Discussion and conclusion are provided in Sections 4 and 5.

## 2. Methods

### 2.1. Model Problem

We consider a model elliptic problem with highly varying conductivity across a thin layer *A* inside *Ω*,

(1) $$\begin{array}{c} -\nabla \cdot \left(\sigma \left(x\right)\nabla u\left(x\right)\right)=0,\;\forall x\in \Omega ,\\ u\left(x\right)=g_{D}\left(x\right),\;\forall x\in \partial \Omega_{D},\\ \frac{\partial u}{\partial n}\left(x\right)=g_{N}\left(x\right),\;\forall x\in \partial \Omega_{N}, \end{array}$$

where the conductivity is given by

(2) $$\begin{array}{c} \sigma \left(x\right)=\{\begin{array}{cc} \sigma_{0}, & x\in \Omega \setminus A,\\ \sigma_{A}, & x\in A, \end{array} \end{array}$$

with the two positive constants *σ*_0_ and *σ*_*A*_, such that *σ*_0_ ≫ *σ*_*A*_. Here, ∂*Ω*_*D*_ and ∂*Ω*_*N*_ denote the parts of the boundary of *Ω* with the Dirichlet and the Neumann boundary conditions, respectively.  Figure 2(a) illustrates the model problem at microscopic scale. In the model, a thin layer *A* with four holes is introduced to simulate a semipermeable membrane of a single cell lying inside an extracellular space *Ω*. Similar model appears in the phantom model invented in [17] based on the experimental phantom adopted by Oh et al.

### 2.2. Multiscalability of Model Problem

 The description of our model inherits two types of multiscales: the conductivity difference between the cell membrane and the extracellular region and the size difference between the thickness of the cell membrane and the diameter of the extracellular region. In order to build a discrete model for such a problem, a very elaborated unstructured mesh is unavoidable to resolve the conductivity discontinuity; see Figure 3. Finite element methods on the given unstructured mesh result in a very ill-conditioned linear system due to highly heterogeneous conductivity, aspect ratio of anisotropic element, and inhomogeneous mesh size [18–21]. In [13], to deal with conductivity discontinuity finite difference methods were developed by correcting the finite difference stencils according to the conductivity discontinuity. Calculation of the correction term is quite complicated and becomes even impossible for such a thin anomaly region *A* with many adopted holes. Therefore both approaches become impractical for our model problem. We emphasize that our purpose is to simulate an apparent conductivity influenced by microscopic structural changes in the cellular membrane. For our purpose, a discrete model with a certain smoothing on the microscopic structures will be more desirable than a very accurate discrete model. In the following subsection, our numerical method will be developed to address this respect.

### 2.3. Model Discretization

 We discretize the model problem in (1) using finite element methods [22] with a uniform mesh. Let *𝒯*_*h*_ be a uniform mesh. We then introduce a piecewise-linear conforming finite element space *X*_*h*_ obtained from the uniform mesh. Here we emphasize that the mesh need not resolve the conductivity discontinuity.

We obtain a weak form of (1) using test functions *v* ∈ *H*_*D*_^1^(*Ω*),

(3) $$\begin{array}{c} \int_{\Omega }\sigma \left(x\right)\nabla u\left(x\right)\nabla v\left(x\right)dx=\int_{\partial \Omega_{N}}g_{N}\left(x\left(s\right)\right)v\left(x\left(s\right)\right)dx\left(s\right), \end{array}$$

where *H*_*D*_^1^(*Ω*) is the space of functions which are square integrable up to first derivatives and have zero values on ∂*Ω*_*D*_, the part of boundary where the Dirichlet boundary condition is given. We then approximate *u*(*x*) with the finite element basis in *X*_*h*_,

(4) $$\begin{array}{c} u\left(x\right)≃\sum_{j\in {𝒩}_{I,N}}U_{j}\phi_{j}\left(x\right)+\sum_{j\in {𝒩}_{D}}U_{j}\phi_{j}\left(x\right). \end{array}$$

Here *ϕ*_*j*_(*x*) are nodal basis functions to nodes *x*_*j*_, *𝒩*_*I*,*N*_ is the set with indices of nodes from the uniform mesh, which are located interior to *Ω* and on ∂*Ω*_*N*_, the part of boundary where the Neumann boundary condition is provided, and *𝒩*_*D*_ is the set with indices of nodes on ∂*Ω*_*D*_, the other part of the boundary where the Dirichlet boundary condition is imposed. For nodes *x*_*j*_ on ∂*Ω*_*D*_, the corresponding nodal values *U*_*j*_ are determined by the Dirichlet boundary condition, that is, *U*_*j*_ = *g*_*D*_(*x*_*j*_) for all *j* in *𝒩*_*D*_.

By approximating *u*(*x*) and using test functions *ϕ*_*i*_(*x*), for all *i* in *𝒩*_*I*,*N*_, we obtain finite element discretization of the model problem in (3),

(5) $$\begin{array}{c} \int_{\Omega }\sigma \left(x\right)\nabla \phi_{i}\left(x\right)\cdot \nabla \left(\sum_{j\in {𝒩}_{I,N}}\phi_{j}\left(x\right)U_{j}\right)dx=g_{i}\;\forall i\in {𝒩}_{I,N}, \end{array}$$

where

(6) gi=∫∂ΩNgN(x(s))ϕi(x(s))dx(s)−∫Ωσ(x)∇ϕi(x)·∇(∑j∈𝒩DgD(xj)ϕj(x))dx.

**Figure 1:**
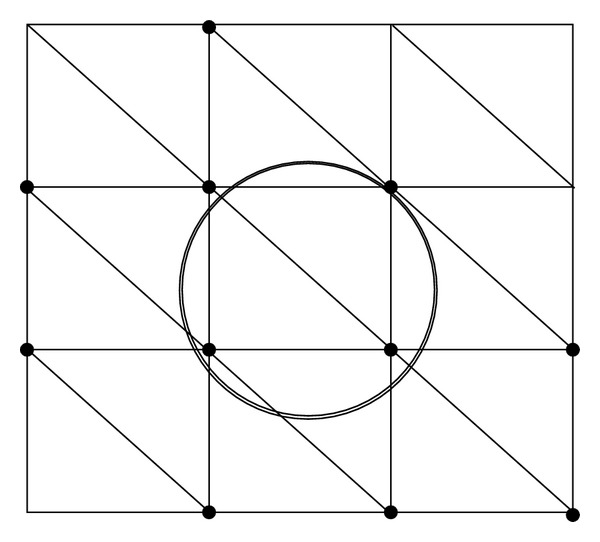
An illustration of uniform mesh and anomaly region: anomaly region *A* is the ring surrounded by the two circles; the microscopic structure of *A* is smoothed out by the nodal basis functions (to the black dots) of which support intersects the anomaly region *A*.

The resulting linear system from the above Galerkin approximation depends on *σ*(*x*). The uniform mesh in our discretization admits the discontinuous conductivity inside a single grid; hence finite element approximation from such uniform mesh results in certain smoothing in our discrete model; see Figure 1. Such a smoothing could provide a more practical tool for calculating the apparent conductivity regarding changes in microscopic cellular structures, while the order of accuracy in our discrete model becomes lower than that in the discrete model from very refined meshes. The size of linear system in our case becomes smaller than the case using a very fine unstructured mesh. However, the conductivity discontinuity across the thin layer still makes the resulting linear system ill conditioned. In order to get faster solutions for the discrete model in (5), we will develop an iterative method for solving the discrete model.

### 2.4. Iterative Method

Our iterative method will be based on a fixed-point iteration. We decompose the conductivity into

(7) $$\begin{array}{c} \sigma \left(x\right)=\sigma_{0}-{\tilde{\sigma }}_{A}\left(x\right), \end{array}$$

where ${\tilde{\sigma }}_{A}(x)$ is defined as

(8) $$\begin{array}{c} {\tilde{\sigma }}_{A}\left(x\right)=\{\begin{array}{cc} 0, & x\in \Omega \setminus A,\\ \sigma_{0}-\sigma_{A}, & x\in A. \end{array} \end{array}$$

Using (7) we rewrite (5) into

(9) σ0∫Ω∇ϕi(x)·∇(∑j∈𝒩I,Nϕj(x)Uj)dx  −∫Ωσ~A(x)∇ϕi(x)·∇(∑j∈𝒩I,Nϕj(x)Uj)dx=gi,

and we obtain

(10) $$\begin{array}{c} \sigma_{0}KU=g+K_{{\tilde{\sigma }}_{A}}U, \end{array}$$

where

(11) (K)ij=∫Ω∇ϕi(x)·∇ϕj(x)  dx, i,j  ∈𝒩I,N,(Kσ~A)ij =∫Ω(σ0−σA)χA(x)∇ϕi(x)·∇ϕj(x)  dx, i,j∈𝒩I,N.

Here *U* and *g* denote the vector of components *U*_*i*_ and *g*_*i*_ for *i* in *𝒩*_*I*,*N*_, respectively, and *χ*_*A*_(*x*) is the characteristic function regarding the set *A*, that is,

(12) $$\begin{array}{c} \chi_{A}\left(x\right)=\{\begin{array}{cc} 1, & x\in A,\\ 0, & x\in \Omega \setminus A. \end{array} \end{array}$$

We now propose an iterative method for (10).

**Algorithm 1 (iterative method)**

 

- Step 1: let *U* ^(0)^ be an initial.
- Step 2: iterate until *U* ^(*n*)^ converges.

Given *U* ^(*n*)^, update *U* ^(*n*+1)^ from

(13) $$\begin{array}{c} \sigma_{0}KU^{\left(n+1\right)}=g+K_{{\tilde{\sigma }}_{A}}U^{\left(n\right)}. \end{array}$$

Before we discuss the convergence of the above iterative method, we define the following concept. For a *m* × *m* matrix *K*_1_, we define a norm by

(14) $$\begin{array}{c} ||K_{1}||\;\;:=\underset{v\in {\mathbb{R}}^{m},\;v\neq 0}{\max}\sqrt{\frac{{\left(K_{1}v\right)}^{T}K_{1}v}{v^{T}v}}. \end{array}$$

We say a matrix *K*_1_ is *symmetric* when *K*_1_^*T*^ = *K*_1_. For symmetric matrices *K*_1_ and *K*_2_, we define the relation

(15) $$\begin{array}{c} K_{1}\leq K_{2}, \end{array}$$

when the two matrices satisfy

(16) $$\begin{array}{c} v^{T}K_{1}v\leq v^{T}K_{2}v,\;\forall v\in {\mathbb{R}}^{m}. \end{array}$$

The relation means that all the eigenvalues of *K*_1_ are bounded by the maximum eigenvalue of *K*_2_. We say that a symmetric matrix *K*_1_ is *positive definite* when

(17) $$\begin{array}{c} v^{T}K_{1}v>0,\;\forall v\left(\neq 0\right)\in {\mathbb{R}}^{m}. \end{array}$$

For a symmetric and positive definite matrix *K*_1_, the norm ||*K*_1_|| is identical to the maximum eigenvalue of the matrix *K*_1_. We note that the two matrices *K* and $K_{{\tilde{\sigma }}_{A}}$ in the above algorithm are symmetric and positive definite. For any given symmetric and positive definite matrix *S*, we obtain that

(18) $$\begin{array}{c} K_{1}\leq K_{2}\;\text{implies}\;\;||S^{-1}K_{1}||\leq ||S^{-1}K_{2}||. \end{array}$$

Since *σ*_0_ > *σ*_*A*_, the matrix $K_{{\tilde{\sigma }}_{A}}$ satisfies that

(19) $$\begin{array}{c} 0<K_{{\tilde{\sigma }}_{A}}<\sigma_{0}K. \end{array}$$

Let *E* ^(*n*+1)^ = *U* ^(*n*+1)^ − *U* ^(*n*)^ and then

(20) $$\begin{array}{c} E^{\left(n+1\right)}={\left(\sigma_{0}K\right)}^{-1}K_{{\tilde{\sigma }}_{A}}E^{\left(n\right)}. \end{array}$$

From (19) combined with (18), we obtain that

(21) $$\begin{array}{c} ||{\left(\sigma_{0}K\right)}^{-1}K_{{\tilde{\sigma }}_{A}}||<1, \end{array}$$

and ||*E* ^(*n*+1)^|| then converge to zero; in other words, the iterates *U* ^(*n*)^ converge,

(22) $$\begin{array}{c} U^{\left(n+1\right)}={\left(\sigma_{0}K\right)}^{-1}g+{\left(\sigma_{0}K\right)}^{-1}K_{{\tilde{\sigma }}_{A}}U^{\left(n\right)}. \end{array}$$

Therefore, our iterative method is a form of a fixed-point iteration.

We also observe fast convergence when the area of *A* is relatively small part of *Ω*, which is the case in our model. At each iteration, we solve the system with the stiffness matrix *σ*_0_ *K* and the right-hand side computed from the previous iterate *U* ^(*n*)^. Since *K* is obtained from the uniform mesh, we can employ any available fast solver to find the update *U* ^(*n*+1)^, such as a multigrid preconditioner or fast Fourier transform. The conductivity discontinuity is treated in the term $K_{{\tilde{\sigma }}_{A}}U^{\left(n\right)}$, which amounts to evaluate integration over the anomaly region *A*. For an accurate integration, we apply the composite Gaussian quadrature.

Our resulting method becomes similar to the immersed boundary methods [14] in the respect that the conductivity discontinuity in the model is treated as the source term of the simple model problem with a uniform conductivity, of which problem is well approximated by using a uniform mesh.

### 2.5. Convergence of Iterative Method

 In this subsection, we will provide a more precise contraction modulus for ${\left(\sigma_{0}K\right)}^{-1}K_{{\tilde{\sigma }}_{A}}$ depending on the relative ratio of the thickness of the anomaly to the mesh size and the relative ratio of *σ*_*A*_ to *σ*_0_. We define

(23) $$\begin{array}{c} \gamma :=\underset{\tau \in {𝒯}_{h}}{\max}\frac{\left|A⋂\tau \right|}{\left|\tau \right|},\;\;\;\;\epsilon :=\frac{\sigma_{A}}{\sigma_{0}}, \end{array}$$

where |*τ*| is the area of the set *τ*.

For each triangle *τ* in *𝒯*_*h*_, such that *τ*⋂*A* ≠ *∅*, we can extend *A* to *B* so that |*B*⋂*τ* | = *γ* | *τ*| and then we define ${\tilde{\sigma }}_{B}$ by

(24) $$\begin{array}{c} {\tilde{\sigma }}_{B}\left(x\right)=\{\begin{array}{cc} \sigma_{0}-\sigma_{A}, & x\in B,\\ 0, & x\in \Omega \setminus B. \end{array} \end{array}$$

Since

(25) $$\begin{array}{c} {\tilde{\sigma }}_{A}\left(x\right)\leq {\tilde{\sigma }}_{B}\left(x\right)<\sigma_{0}, \end{array}$$

we obtain that

(26) $$\begin{array}{c} K_{{\tilde{\sigma }}_{A}}\leq K_{{\tilde{\sigma }}_{B}}<\sigma_{0}K, \end{array}$$

where matrices $K_{{\tilde{\sigma }}_{A}}$, $K_{{\tilde{\sigma }}_{B}}$, and *σ*_0_ *K* correspond to conductivity functions ${\tilde{\sigma }}_{A}(x)$, ${\tilde{\sigma }}_{B}(x)$, and *σ*_0_, respectively.

By using that |*B*⋂*τ*| is the same value *γ* | *τ*| for all triangles *τ*, which intersect *A*, and (∇*ϕ*_*i*_(*x*)·∇*ϕ*_*j*_(*x*))|_*τ*_ are constant for each *τ*, we have

(27) $$\begin{array}{c} K_{{\tilde{\sigma }}_{B}}\leq \gamma \left(\sigma_{0}-\sigma_{A}\right)K \end{array}$$

and thus

(28) $$\begin{array}{c} K_{{\tilde{\sigma }}_{A}}\leq \gamma \left(\sigma_{0}-\sigma_{A}\right)K. \end{array}$$

By applying (*σ*_0_ *K*)^−1^ on both sides of the above inequality, see (18), we finally obtain that

(29) $$\begin{array}{c} ||{\left(\sigma_{0}K\right)}^{-1}K_{{\tilde{\sigma }}_{A}}||\leq \gamma \left(1-\epsilon \right). \end{array}$$

The error reduction in the iterative method is bounded by *γ* and *ϵ*. When *A* intersects only a small number of triangles in *𝒯*_*h*_, we obtain a better reduction factor since most entries in $K_{{\tilde{\sigma }}_{A}}$ are zero.

**Theorem 2**

The error reduction factor in the iterative method is at least determined by

(30) $$\begin{array}{c} {\left(\sigma_{0}K\right)}^{-1}K_{{\tilde{\sigma }}_{A}}\leq \gamma \left(1-\epsilon \right), \end{array}$$

where *γ* and *ϵ* are parameters defined by

(31) $$\begin{array}{c} \gamma :=\underset{\tau \in {𝒯}_{h}}{\max}\frac{\left|A⋂\tau \right|}{\left|\tau \right|},\;\;\;\epsilon :=\frac{\sigma_{A}}{\sigma_{0}}. \end{array}$$

**Remark 3**

When the thickness of *A* is *δ*, *γ*≃(*δ*/*h*). Therefore the error reduction factor is determined by the relative ratio of the thickness of the anomaly to the mesh size. As the anomaly region *A* becomes thinner and occupies a smaller part of *Ω*, the error reduction factor becomes smaller for a given mesh size *h* and a given relative ratio of conductivity *ϵ*.

**Remark 4**

For a faster convergence of our iterative method, the suggested mesh size *h* for a given thickness *δ* is to satisfy

(32) $$\begin{array}{c} h>\frac{1}{c}\delta , \end{array}$$

for some positive number *c* < 1. With such an *h*, the error reduction factor is then bounded by the constant *c*. From the following numerical experiments, the *L* ^2^ errors of solutions in our discrete model are observed to follow *O*(*h*). The mesh size *h* should be determined considering the required accuracy as well as the convergence of the iterative method.

## 3. Numerical Results

**Figure 2:**
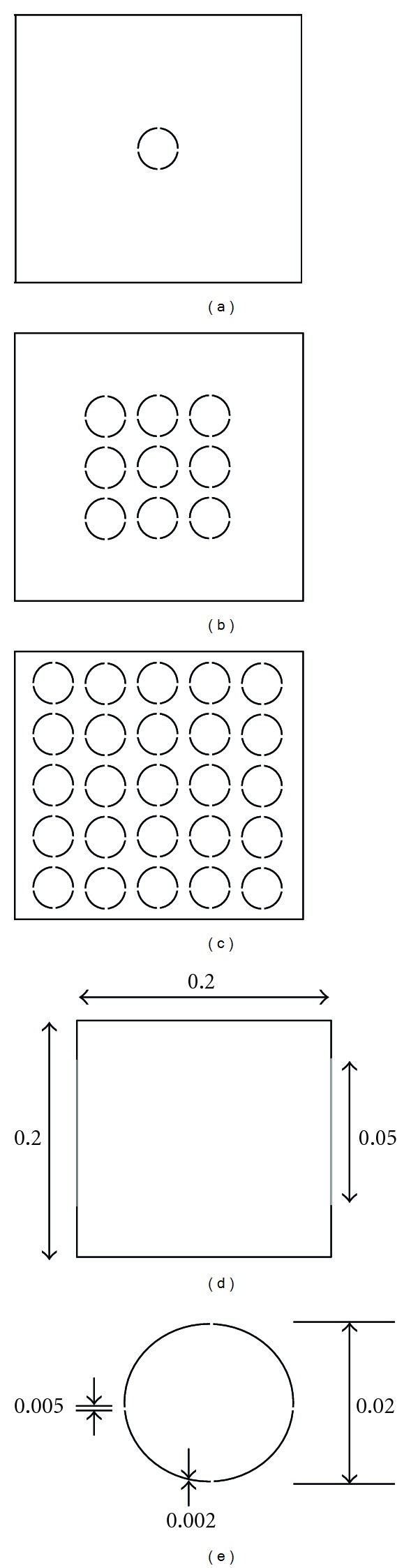
Model geometry with (a) 1, (b) 9, and (c) 25 anomalies with four holes orthogonal to each other. (d) Outer domain dimensions and (e) anomaly dimensions.

We present numerical experiments on the proposed method. We will consider models in Figure 2. The problem domain *Ω* = (−0.1  0.1)^2^ is a rectangular region and the anomaly region *A* consists of thin circles with small adopted holes. The thickness *δ* of the circle is 0.002 and the diameter of the each circle is 0.02. The diameter of holes introduced in each circle is 0.005. The conductivity in the anomaly region is given by 0.001 and the conductivity is given by 1 elsewhere, that is, *σ*_*A*_ = 0.001 and *σ*_0_ = 1. For all the models in the following experiments, *ϵ* = 0.001, *δ* = 0.002, and *γ* ( = *δ*/*h*) will be determined once the mesh size *h* is chosen.

**Figure 3:**
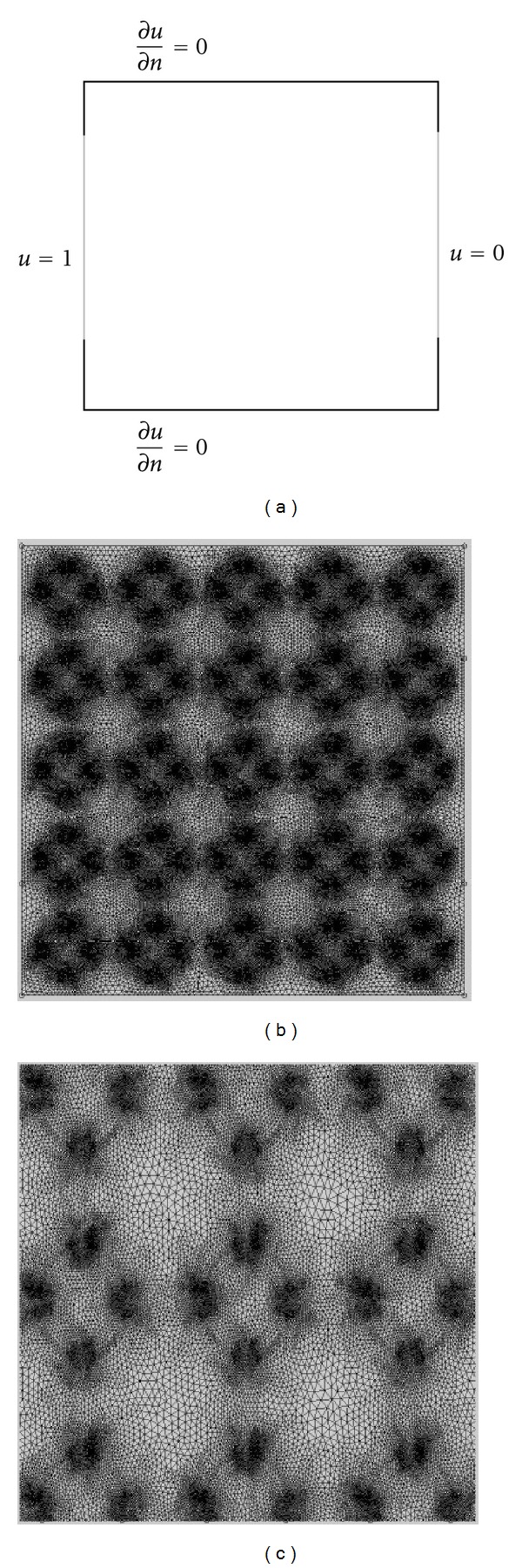
Numerical modeling of problem in COMSOL. (a) Boundary conditions, (b) mesh, and (c) magnified domain.

We study the behavior of errors by approximating the model problem with finite element methods on uniform grids, which do not resolve conductivity discontinuity across the anomaly region *A*. We first study the single cell model shown in Figure 2(a). We then consider more complex models with multiple cells as shown in Figures 2(b) and 2(c). In order to compute errors, we obtain a reference solution from COMSOL by solving the same model problems using a very refined unstructured mesh which can resolve the conductivity discontinuity; see Figure 3. We considered the stationary solver of COMSOL to obtain the reference solution for various models in this work. The stationary solver works on linear and nonlinear stationary PDE problems. Internally, a function called femstatic works as a stationary solver for both linear and nonlinear problems. The default value is “auto,” which means that femstatic automatically selects a solver depending on the problem's linearity. A linear solver is selected by femstatic for the model problems in this work. COMSOL uses either direct or iterative linear system solvers to solve the system matrix. Various preconditioner algorithms are used to deal with the ill conditioning of the system matrix. Those include incomplete LU, geometric multigrid, incomplete Cholesky, and few others (COMSOL Inc., USA).

In Table 1, we report relative *L* ^2^ errors of the solution (voltage) and the current density for decreasing the mesh size *h*. Here *N* denotes the number of grid in each direction and *Ω*_*I*_ denotes that the error is computed over the region inside the circle. For example, the relative *L* ^2^ error for *u* − *U*_*h*_ in *Ω*_*I*_ is calculated by

(33) $$\begin{array}{c} {||u-U_{h}||}_{\Omega_{I}}\;:=\sqrt{\frac{\sum_{x_{i}\in \Omega_{I}}^{}{\left(u\left(x_{i}\right)-U_{h}\left(x_{i}\right)\right)}^{2}}{\sum_{x_{i}\in \Omega_{I}}^{}u^{2}\left(x_{i}\right)}}, \end{array}$$

where *u* is the reference solution from COMSOL, *U*_*h*_ is the solution from our method with mesh size *h*, and *x*_*i*_ are grid points located in *Ω*_*I*_. In the iterative method, the iteration is stopped when the relative error in two consecutive iterates is reduced by a factor of 10^6^, that is,

(34) $$\begin{array}{c} \frac{{||U^{\left(n+1\right)}-U^{\left(n\right)}||}_{\Omega }}{{||U^{\left(n\right)}||}_{\Omega }}\leq 10^{-6}. \end{array}$$

and the number of iteration counts is also reported in Table 1 for each grid level.

We observe that the errors in voltage follow *O*(*h*) over the whole domain *Ω* and the errors inside the circle show better accuracy. The current density *J* = ||(*J*_1_, *J*_2_)|| is calculated at each grid point (*x*_*i*_, *y*_*j*_) by using the voltage solution *u*. The current density *J*_*h*_ from our method is calculated as the same way using *U*_*h*_. As we can see from the results, the errors in the current density become much smaller as the mesh size is getting smaller, since the derivative of voltage is well approximated using the smaller mesh size. To obtain more accurate approximation for the current density, we can formulate a first-order system of (1) by introducing a new variable, **J** = *σ*(*x*)∇*u*(*x*). A similar idea to the current work can be used for the first-order system. This problem will be addressed in our forthcoming research.

About the iteration counts, the error reduction rate in the iterative method depends on *γ* and *ϵ*. For a given *N*, *h* = 0.4/*N* and then the ratio between *h* and *δ* = 0.002 becomes

(35) $$\begin{array}{c} \gamma ≃\frac{\delta }{h}=0.005\times N. \end{array}$$

As we can observe from the numerical experiments when *N* < 100, we have faster convergence in the iterative method. 

**Figure 4:**
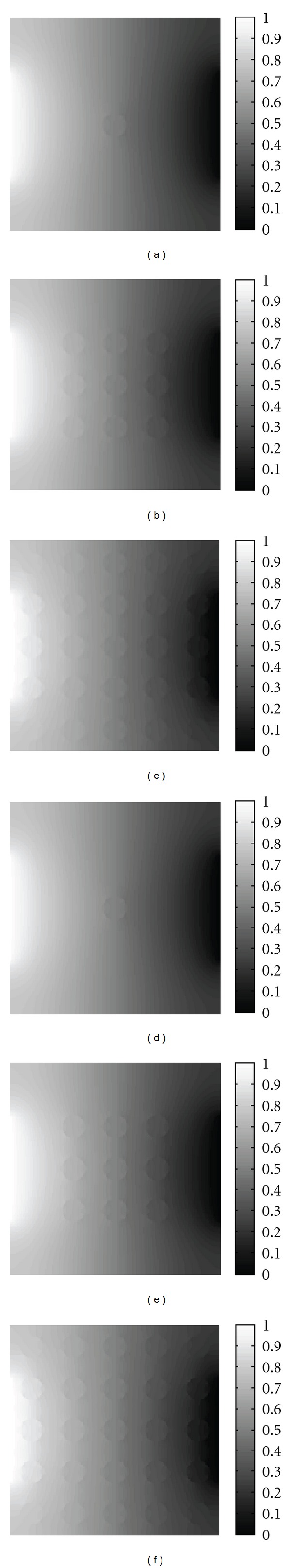
(a)–(c) are reference solutions from the model with 1, 9, and 25 anomalies with 4 holes. (d)–(f) are the corresponding solutions using the iterative method. The resolution is 256 × 256.

**Figure 5:**
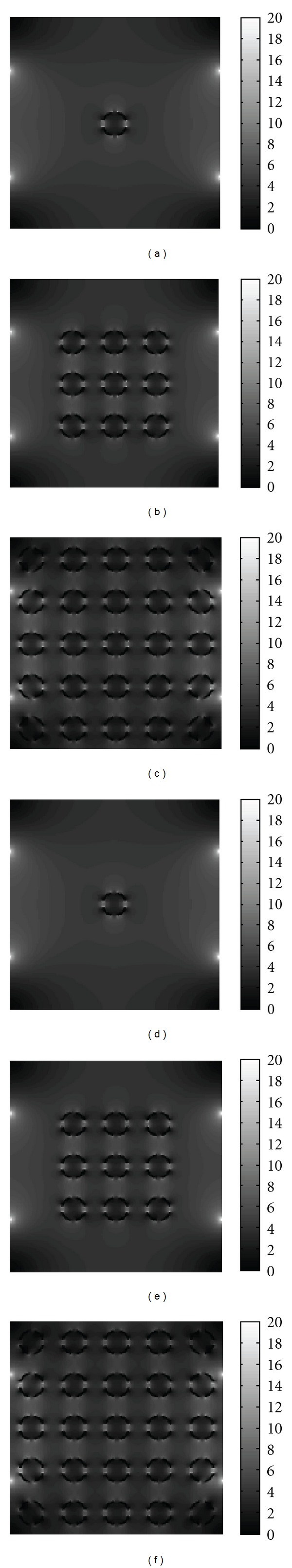
(a)–(c) are reference current densities from the model with 1, 9, and 25 anomalies with 4 holes. (d)–(f) are the corresponding current densities using the iterative method. The resolution is 256 × 256.

 In Table 2, we report errors for models with a more complicated anomaly *A*, which consists of many circles with holes. The behavior of errors is similar to this observed in Table 1. For the same grid level *N*, the iteration count gets larger as more circles are introduced in the anomaly region *A*, that is, from model (a) to model (c). When *N* ≤ 128, even for a very complicated anomaly case of model (c) we observe quite good iteration counts. In Figures 4 and 5, we also plot solutions and current densities obtained from COMSOL and our method and we can observe good agreement for all the three models. 

In order to show that our method is capable of capturing macroscopic properties, we apply our method to a model with anisotropic conductivity. Here we consider a circle with two horizontally adopted holes; the center of each hole is located at the left and right end points of the circle. To study anisotropic models, we inject a current in the horizontal direction or in the vertical direction, and we also consider a circle without any adopted hole.

**Figure 6:**
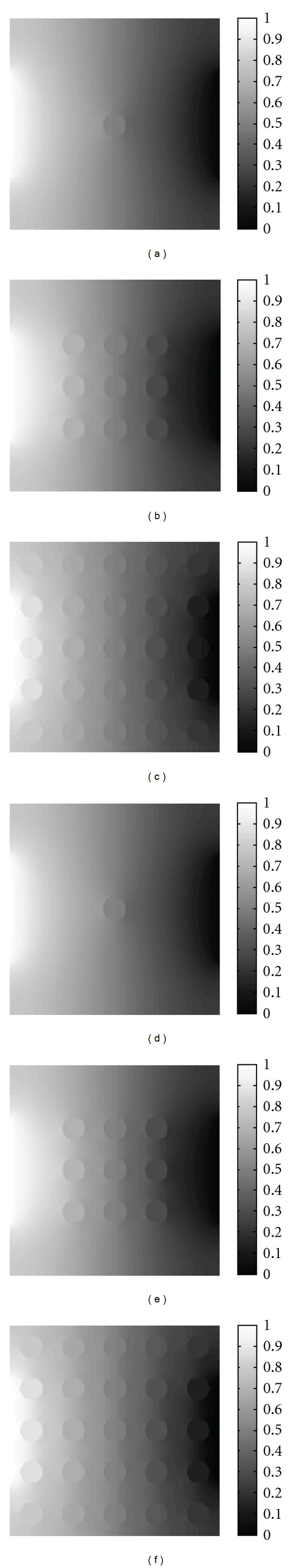
Horizontal current injection for horizontally adopted holes: (a)–(c) are reference solutions from the model with 1, 9, and 25 anomalies with 2 holes. (d)–(f) are the corresponding solutions using the iterative method. The resolution is 256 × 256.

**Figure 7:**
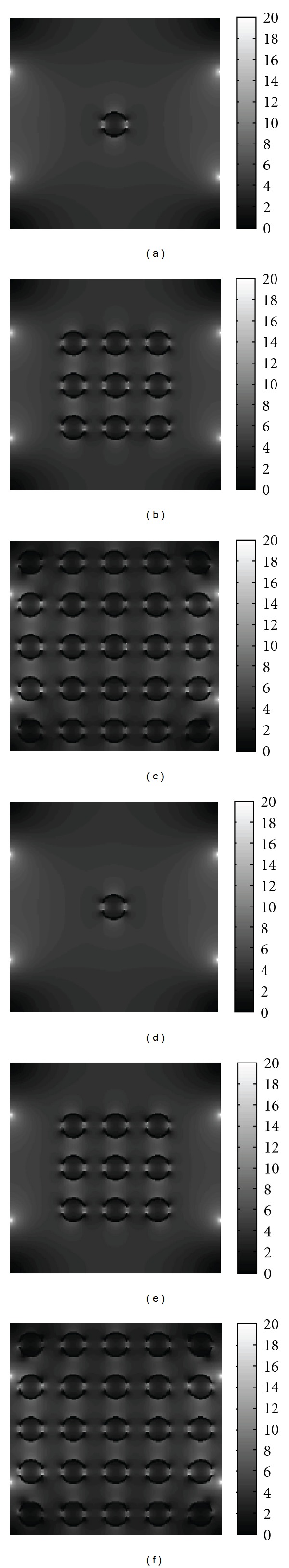
Horizontal current injection for horizontally adopted holes: (a)–(c) are reference current densities from the model with 1, 9, and 25 anomalies with 2 holes. (d)–(f) are the corresponding current densities using the iterative method. The resolution is 256 × 256.

In Figures 6 and 7, the voltage and current density are presented for models with each anomaly consisting of circles with two horizontally adopted holes. Here on the boundary of the domain the current is injected in the horizontal direction. We compare the results from our method with *N* = 256 and from COMSOL with a very fine mesh. We observe that these two results match well and they are also in a good agreement with those in the previous two Figures 4 and 5, when the anomaly consists of circles with two horizontal and two vertical holes, and the current is horizontally injected on the boundary.

**Figure 8:**
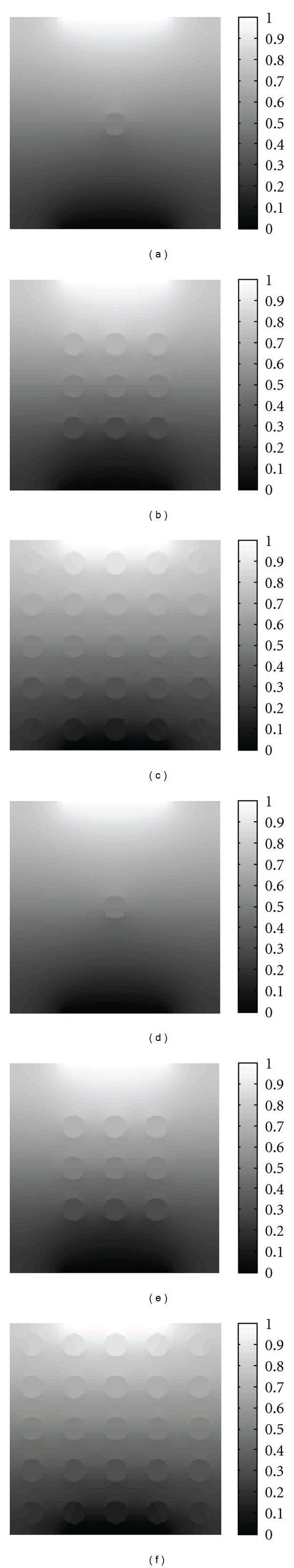
Vertical current injection for horizontally adopted holes: (a)–(c) are reference solutions from the model with 1, 9, and 25 anomalies with 2 holes. (d)–(f) are the corresponding solutions using the iterative method. The resolution is 256 × 256.

**Figure 9:**
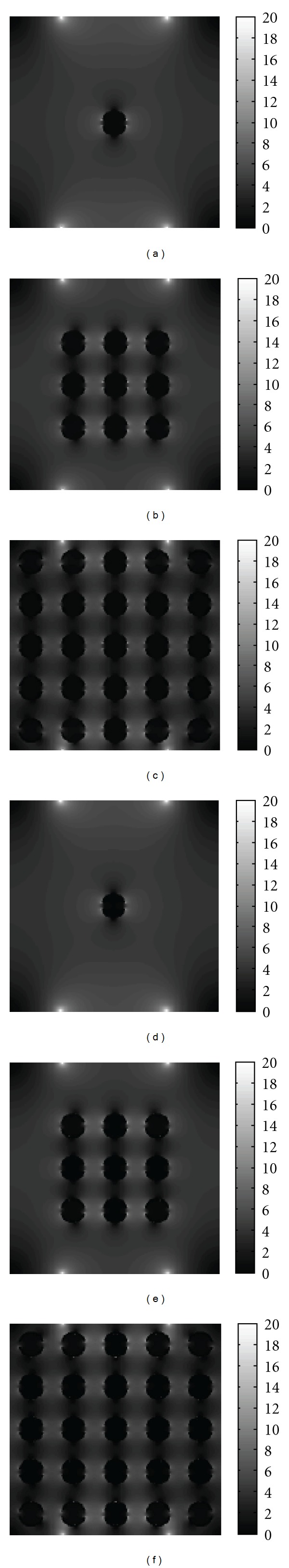
Vertical current injection for horizontally adopted holes: (a)–(c) are reference current densities from the model with 1, 9, and 25 anomalies with 2 holes. (d)–(f) are the corresponding current densities using the iterative method. The resolution is 256 × 256.

In Figures 8 and 9, the voltage and current density are presented for models with each anomaly consisting of circles with two horizontally adopted holes. Here on the boundary of the domain the current is injected in the vertical direction. We compare the results from our model with *N* = 256 and from COMSOL with a very fine mesh. We observe that the vertically injected current cannot detect the horizontally adopted holes in each circle.

**Figure 10:**
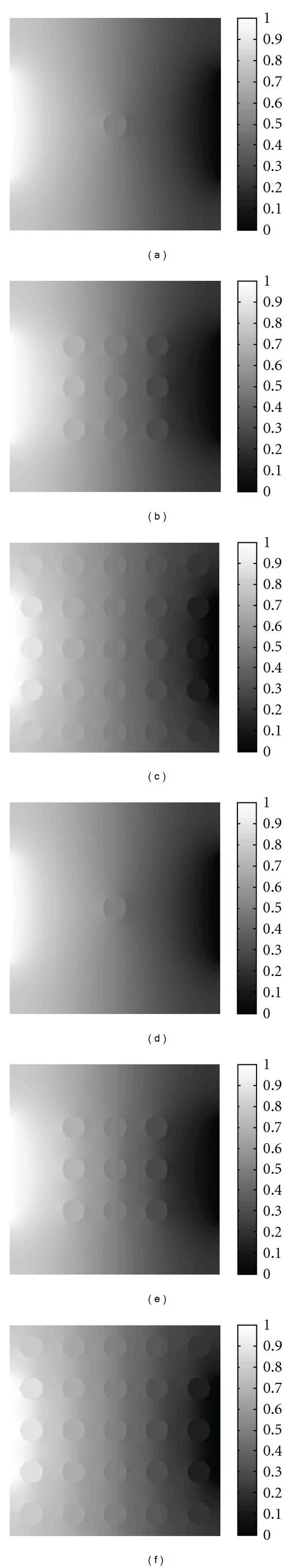
Horizontal current injection for no adopted holes: (a)–(c) are reference solutions from the model with 1, 9, and 25 anomalies with no holes. (d)–(f) are the corresponding solutions using the iterative method. The resolution is 256 × 256.

**Figure 11:**
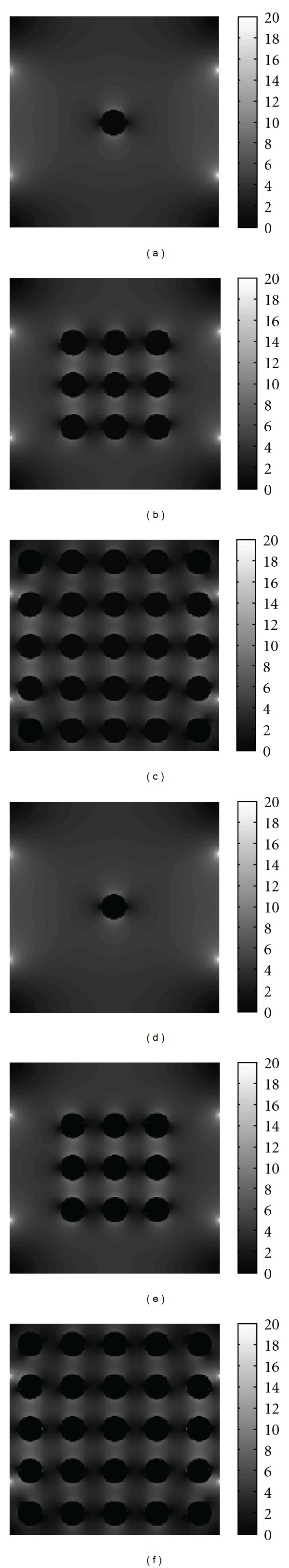
Horizontal current injection for no adopted holes: (a)–(c) are reference current densities from the model with 1, 9, and 25 anomalies with no holes. (d)–(f) are the corresponding current densities using the iterative method. The resolution is 256 × 256.

In Figures 10 and 11, the voltage and current density are presented for models with each anomaly consisting of circles without any adopted holes. We compare the results from our model with *N* = 256 and from COMSOL with a very fine mesh. We observe that the results here are quite similar to those in Figures 8 and 9.

The numerical study on anisotropic conductivity models presents that our method is capable of capturing the macroscopic conductivity with respect to changes in microscopic cellular membranes.

## 4. Discussion

 We developed a practical numerical method for simulating the macroscopic conductivity related to microscopic changes in cellular membranes. Finite element discretization with a uniform mesh is applied to the multiscale model without much concern on the microscopic structures. We refer to previous studies as those in [12, 14] where similar ideas were developed for multiscale problems or for multiple structures.

For a more practical method, we used a standard linear finite element basis of the uniform mesh rather than using the coarse basis in [12]. Similarly to [14], smoothed approximation of microscopic cellular structures is imbedded in the right-hand side of the iterative method, where a simple discrete model with uniform conductivity is solved to improve the accuracy. Convergence of the iterative method was analyzed regarding the contrast in the conductivity difference and the relative ratio of the cell membrane to the mesh size.

Since the uniform mesh does not resolve the conductivity discontinuity, our method results in a less accurate approximation as in [14]. In the current work, we report numerical results which present *O*(*h*) convergence in *L* ^2^ errors in the whole domain *Ω*. Such property of the approximation was already reported in [14] when a very thin elastic body is considered.

From numerical experiments, we can see that our method is capable of capturing the apparent conductivity with respect to changes in microscopic cellular structures. To obtain a more accurate current approximation, our method can be further applied to the first-order system of two unknowns, **J** = *σ*(*x*)∇*u* and *u*, and this will be addressed in our forthcoming research.

## 5. Conclusion

 This kind of multiscale approach is needed to properly interpret reconstructed conductivity images in MREIT in relation with microscopic structural changes in cellular membranes. We will use the proposed method to construct an inhomogeneous tissue model including many cells with different membrane structures of holes. We will compute magnetic flux density as well as voltage and current density for MREIT simulation. Using the computed magnetic flux density, we will reconstruct images of apparent conductivity. We will see how apparent conductivity changes as we change the microscopic cellular membrane structures. We will combine the proposed method with MREIT simulation so that we can interpret an apparent conductivity reconstructed by using an MREIT algorithm.

## Figures and Tables

**Table 1:** The *L* ^2^ errors for the model (a). Ω: the whole domain, Ω_*I*_: the region inside the circle, ||*u*−*U*_*h*_||_*D*_: errors of voltage (solution) in the region *D*, ||*J*−*J*_*h*_||_*D*_: errors of current density (computed from voltage) in the region *D*, and Iter.: number of iterations.

*N*	||*u*−*U*_*h*_||_Ω_	||*u*−*U*_*h*_||_Ω_*I*__	||*J*−*J*_*h*_||_Ω_	||*J*−*J*_*h*_||_Ω_*I*__	Iter.
16	0.0081	0.0000	0.0692	0.0157	4
32	0.0046	0.0145	0.0692	0.5800	6
64	0.0033	0.0070	0.0548	0.5347	10
128	0.0017	0.0038	0.0340	0.2208	38
256	0.0007	0.0001	0.0324	0.0061	164

**Table 2:** The *L* ^2^ errors for the models (b) and (c). ||*u*−*U*_*h*_||_Ω_: errors in the solution (voltage), ||*J*−*J*_*h*_||_Ω_: errors in current density (computed from voltage), and Iter.: number of iterations.

*N*	Model (b)			Model (c)		
	||*u*−*U*_*h*_||_Ω_	||*J*−*J*_*h*_||_Ω_	Iter.	||*u*−*U*_*h*_||_Ω_	||*J*−*J*_*h*_||_Ω_	Iter.
32	0.0121	0.1828	7	0.0096	0.3220	7
64	0.0120	0.1800	13	0.0091	0.3070	14
128	0.0067	0.1413	47	0.0053	0.2449	50
256	0.0022	0.0462	397	0.0035	0.0640	739
